# Agitation role (Dissolved Oxygen) in production of laccase from newly identified *Ganoderma multistipitatum* sp. nov. and its effect on mycelium morphology

**DOI:** 10.1186/s12866-023-03009-2

**Published:** 2023-10-02

**Authors:** Aisha Umar, Islem Abid, Mohamed S Elshikh, Laurent Dufossé, Ahmed M. Abdel-Azeem, Iftikhar Ali

**Affiliations:** 1 Institute of Botany, University of the Punjab, Lahore, Pakistan; 2 Department of Botany and Microbiology, College of Science, King Saud University, P.O. 2455, Riyadh, 11451 Saudi Arabia; 3 Laboratoire CHEMBIOPRO (Chimie et Biotechnologie des Produits Naturels), ESIROI Département agroalimentaire, Université de La Réunion, 15 avenue René Cassin Saint-Denis, 97490, France; 4 Botany and Microbiology Department, Faculty of Science, Suez Canal University, Ismailia, 41522, Egypt; 5 Department of Genetics, Faculty of Natural and Agricultural Sciences, University of the Free State, Bloemfontein, 9300, Republic of South Africa; 6 Department of Genetics and Development, Columbia University Irving Medical Center, New York, NY 10032, USA; 7 School of Life Sciences & Center of Novel Biomaterials, The Chinese University of Hong Kong, Shatin, Hong Kong

**Keywords:** Shear, Rpm, Aerobic, Submerge, Dissolved oxygen

## Abstract

**Background:**

Agitation speed influenced the production rate of laccase. Orbital speed not only influenced the enzyme production, but was also effective to dissolve the oxygen during growth of mycelium, spores, and chlamydospores. Shear effects of speed greatly influenced the morphology of mycelium.

**Methods:**

*Ganoderma multistipitatum* was identified by ITS marker. Phylogenetic tree was constructed for species identification. Qualitatively by plate method contained guaiacol indicator, while quantitatively by submerged fermentation and Central Composite Design applied on agitation parameter for maximum laccase potential of this species. The effects of agitation speed on mycelium morphology were observed under compound and scanning electron microscope.

**Results:**

Statistical optimization of agitation conditions were performed by using response surface methodology to enhance the production of laccase from *Ganoderma multistipitatum* sp. nov. Maximum laccase yield (19.44 × 10^5^ ± 0.28 U/L) was obtained at 150 rpm grown culture, which was higher than predicted value of laccase production (19.18 × 10^5^ U/L) under aerobic conditions (150 rpm). The 150 rpm provided the continuous flush of oxygen. The DO (dissolved oxygen) was maximum (65%) for “27 h” incubation at 150 rpm during laccase synthesis. The statistical value of laccase production was minimum under anaerobic or nearly static condition of 50 rpm. The predicted (12.78 × 10^5^ U/L) and obtained (12.82 × 10^5^ U/L) yield was low at 50 rpm. Optimization of orbital shaking for aeration conditions were performed by the use of “Response Surface Methodology”. The submerged shaking flasks were utilized as a nutrients growth medium to maximize the production of laccase from *G. multistipitatum*. The minimum incubation time highly influenced the laccase yield from 7 to 15 days via utilization of less cost-effective medium under a promising and eco-friendly method. The morphological effects of rpm on mycelium were examined under compound and scanning electron microscopy. Higher rpm (200, 230) shear the mycelium, while 150 to 200 rpm exhibited smoother and highly dense branches of mycelia.

**Conclusion:**

The shear forces of 200 rpm caused the damages of mycelium and cells autolysis with less laccase production. This study concluded that 150 rpm saved the life of mycelium and enhanced the production rate of enzymes.

## Introduction

*Ganoderma* a basidiomycete and cosmopolitan genus belongs to family Ganodermataceae. Laccase exhibit versatile potential in multiple applications like biofuel production, dye degradation, biosensor, natural fiber extraction, bioremediation and effluent treatment [1, 2]. *Ganoderma* species are considered the promising candidates in sense of laccase production at industrial, environmental, biotechnological and healthcare sectors [3]. This health oriented herbal mushroom comprised numerous bioactive secondary compounds important in pharmacology due to its therapeutic effects [4]. Laccase distribute within various organs and tissues of living organisms and play important role in anabolism [5, 6]. So, subcellular localized substrates are required for the laccase to perform physiological process of the human beings. The formation of intracellular biopolymers by laccase affect the normal metabolism, physiological, and biochemical attributes of the organism [7].

Laccase contribute in formation of melanin pigments in fungal cell walls [8], because melanogenesis is restricted to certain developmental stages of mycelium, cell wall pigmentation, and fruiting body formation [9]. Basidiomycotal laccases are involved in decomposition of lignocellulose polymers, defense/protection, virulence, pathogenesis, pigmentation, and sporulation processes. In fungi, laccase formulated melanin contribute in resistance against hydrolytic enzymes and play protective role in spores against environmental stresses (harmful UV radiation, ROS, and toxic heavy metals) [10].

Currently, to fulfil the demands of commercial applications, the sufficient quantity of the laccase is not available. These demands can be fulfilled via use of different strategies like optimization of ecofriendly strategy at submerge fermentation level. Ganodermal laccase of this work has advantages in functioning and stability over a wide range pH and temperature [4]. The early production owing the cells autolysis during submerged fermentation [11]. This is less cost-effective and industrially sustainable medium for maximum production of laccase yield.

Agitation speed play an important role in nutrients availability to mycelium by mixing the nutrients in shake flask system [12]. The rotational speed influence the enzymes activities in different fungi [13], e.g., agitation supplies (1) mixing, (2) mass, (3) heat transfer, and improving the (4) DO (dissolved oxygen) of culture medium. Consequently, the optimal rotational speed is necessary to estimate the maximum laccase production under the influence of dissolved O_2_. This study also aimed to determine the effects of agitation speed on laccase production and its influence on mycelium of *Ganoderma* in submerged fermentation using shake flask system.

Oxidative stress (imbalance between production and accumulation of ROS) normally generated as by-products of oxygen metabolism, damages the functioning of laccase in fungi [14]. Oxygen provides redox potential to laccase action and energy for cellular activities, because O_2_ acts as a terminal e^−^ acceptor for oxidative reactions. Redox ability of laccase produce secondary metabolites. The requirement of oxygen is different in basidiomycetes. Oxygen promote the sexual differentiation, cleistothecia initials, fungal symbiosis, pathogenesis, and polarised hyphal growth.

### Objective

In this study, the ability of laccase production was evaluated from new *Ganoderma multistipitatum* explored first time under the influence of oxygen supply by orbital shaker at certain range of rpm. The effects on mycelium morphology via supply of oxygen at different rpm were also observed.

## Materials & methods

### Studied material

The specimens (*Ganoderma multistipitatum* sp. nov.) studied here was collected in 2019 from the Botanical Garden of Government College University, Lahore, Pakistan grown on *Pinus roxburghii. Ganoderma multistipitatum* (strains GCB 101,102) were deposited in the Fungarium of Suez Canal University (https://ccinfo.wdcm.org/collection/by_id/1180), at Botany and Microbiology Department, Faculty of Science, Ismailia 41,522, Egypt under accession number SCUF 1055, 1056, respectively.

### DNA extraction

Modified CTAB procedure was followed to extract total genomic DNA from dried specimens [15]. This region was amplified by using primers ITS1 and ITS2 [16]. Reaction mixtures (20 µL) contained 0.5 µL template DNA, 8.5 mL distilled water, 0.5 µL of each primer, and 10 mL PCR mix. Amplification comprised 35 cycles of 95 ºC for 30 s, 52 ºC for 30 s, and 72ºC for 1 min, followed by a final extension at 72 ºC for 10 min. Amplified PCR products were purified and sequenced by TSINGKE Co. Ltd. (China).

### Data analysis by sequence alignment and molecular phylogeny

The ITS data set comprised DNA sequences of specimens from Pakistan and related species. Additionally, ITS sequences of related species were downloaded from GenBank and literature. The phylogenetic tree was constructed to matrix the species in which *Amaurodema rude* selected as an outgroup. All sequences were automatically aligned by MAFFT [17]. The sequences of this species were deposited finally in GenBank for authenticity.

### Qualitative plate analysis

Malt Extract Agar media of pH 5.0 in g/L was prepared by ‘Malt Extract 7, MgSO_4_.7H_2_O 0.5, Agar 10, KH_2_PO_4_ 0.5, K_2_HPO_4_ 0.5, MnSO_4_ 0.05, ZnSO_4_ 0.005, Glucose 15, and Peptone 2.5. Autoclaved at 121 ºC the above broth for 20 min with 0.02% guaiacol to evaluate the laccase production [18]. Plates incubated for 5 days at 30 ºC [4]. The reddish brown oxidation zone in agar plate indicate the laccase secretion from *G. multistipitatum* sp. nov.

### Quantitative analysis and optimization of agitation speed

Kirk’s medium for quantitative laccase activity in shake flasks with nutrients and tracer elements (g/L) were taken for the growth of mycelium. Nutrients augmented in g/1L at pH 5.0 (glucose 10 g, yeast extract 5 g, starch 1 g, while tracers MgSO_4_.7H_2_O 0.5 g, NaCl 0.5 g, FeSO_4_.7H_2_O 0.5 g, KH_2_PO_4_ 0.046 g, K_2_HPO_4_ 0.1 g, CaCl_2_.2H_2_O 0.5 g, ZnSO_4_ 0.02 g, CuSO_4_.5H_2_O 0.5 g, H_4_PO_4_ 1.0 g, Na_4_HPO_4_ 0.05 g, MnSO_4_ 0.001 g, ZnSO_4_ 0.001 g [4, 19] was autoclaved. After cooling, mycelium discs (5 mm diameter) of pure *G. multistipitatum* sp. nov. were inoculated in liquid broth (100 mL) of each flask. These flasks were incubated at 27 °C without shaking for 3 days. Flasks dynamically moved after 3rd day via shaker for optimization the orbital frequency to evaluate the laccase activity.

The enzyme activity was determined by 100 mM guaiacol substrate dissolved in 100 mM sodium acetate buffer (pH 5.0). This reaction mixture contained 1.5 mL acetate buffer, 1.5 mL guaiacol and 1.0 mL of crude enzyme source. The laccase activity was measured at 27 ± 2 °C or room temperature after 15–30 min. The change in absorbance of reaction mixture comprised guaiacol was monitored for 3 min at 470 nm by UV Spectrophotometer [20]. This activity was determined via following formula ($\varDelta \text{A}\text{b}\text{s} 3-5 \text{m}\text{i}\text{n}$) in U/L [21].

$$\frac{{\text{U}}}{{\text{L}}}\, = \,\Delta {\text{Abs470}} * \,\frac{{{\text{Vt}}}}{{\varepsilon * 1 * {\text{Vs}}}}$$

Where,

€ = 6,740 M ^− 1^ cm ^− 1^ extinction coefficient of guaiacol.

Vt = Total vol. of reaction mixture (mL).

Vs = Vol. of the sample (mL).

l = Length of cuvette (1 cm).

Fermentation broth was settled at “50, 100, 150, 200 and 230” rpm to observe the maximum production rate of laccase.

### Statistical optimization of laccase

The oxygen factor has dominant effect on laccase secretion, which further optimized by statistical experimental design, i.e., “Response Surface Methodology” [22]. This design was applied on three significant factors like (1) Agitation rate, (2) Agitation time and (3) Stationary time for response surface mapping [23] and the optimization of laccase from *Ganoderma* species. Central Composite Design (CCD) with three significant factors at five levels was implemented to investigate the 1st and higher-order of each factor effects and linkage in them. All the experiments were examined in triplicates. The statistical software package Design-Expert® (version 10.0.5) was used to generate 3D graphs.

1 $$LA\, = \,\beta 0\, + \,\sum \beta iXi\, + \,\sum \beta iiXi2\, + \,\sum \beta ijXi$$

where.

(LA is predicted response, *β0* is offset term, *βi* is linear effect, *βii* is squared effect, *βij* is interaction effect and *Xi* is the dimensionless coded value of the independent variables under study).

#### Compound microscopy

Microscopic structures observed from cross section of basidiome that was soaked in KOH (5%), stained with Congo red (1%), and viewed under a MX4300H compound light microscope (Meiji Techo Co., Ltd., Japan). Data of anatomical features were recorded from at least 30 measurements of face view and side view at a magnification of 100X.

### Scanning electron microscopy

The 7 day old ganodermal mycelia were taken from several agitation speed (50, 100, 150, 200 and 250 rpm). Mycelia were washed 2–3 times with distilled water and then blotted in dry Whatman’s filter papers. The samples for SEM were prepared accordingly Darah et al. [24] and observed under FESEM LEO Supra 50VP, Carl Zeiss, Germany.

### Determination of dissolved oxygen (DO) concentration

The DO was measured by serializable dissolved oxygen sensor (Mettler Toledo) coupled to the bioreactor (Labfore; capacity: 3 L). This sensor was calibrated by the use of sodium sulphite method [25].

### Statistical analysis

The data collected from various parameters during presented study was subjected to statistical analysis. The complete experiment was conducted in triplicate and the values were presented in mean ± standard deviation.

## Results

### Phylogenetic inference

**Fig. 1:**
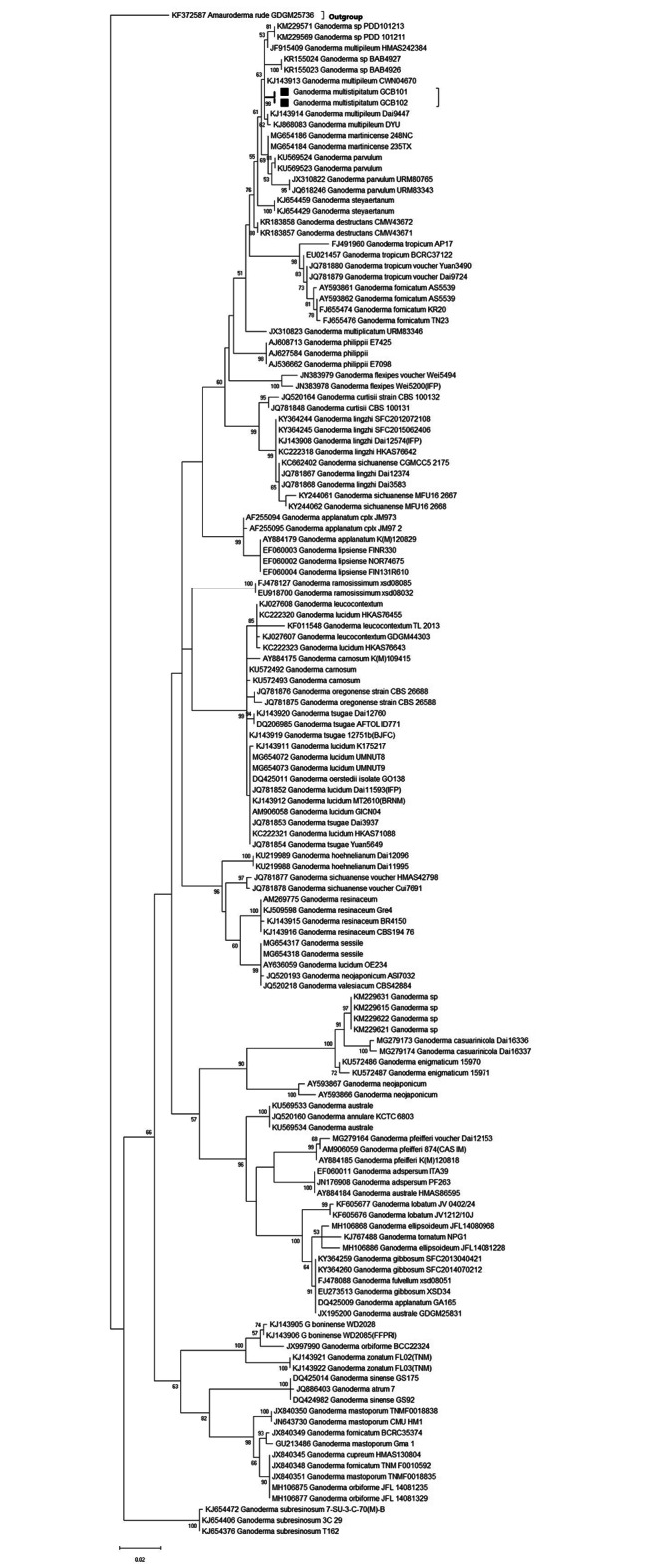
Phylogenetic tree of *Ganoderma multistipitatum* (New species represented by black dot and box)

ITS sequences (GenBank No. ON032992, ON032991) of *G. multistipitatum* with *Amaurodema rude* (outgroup) was analyzed by ML method. Reference sequences from GenBank of closely related consensus were also combined in phylogeny. The alignment was done by online MAFFT program [17]. ML analysis with statistical bootstrap (99%) was presented in Fig. 1. Molecular phylogeny of this study indicated the *G. multistipitatum* was a new species closely matrixed to *Ganoderma* clade [26].

### Preliminary qualitative laccase production

**Fig. 2:**
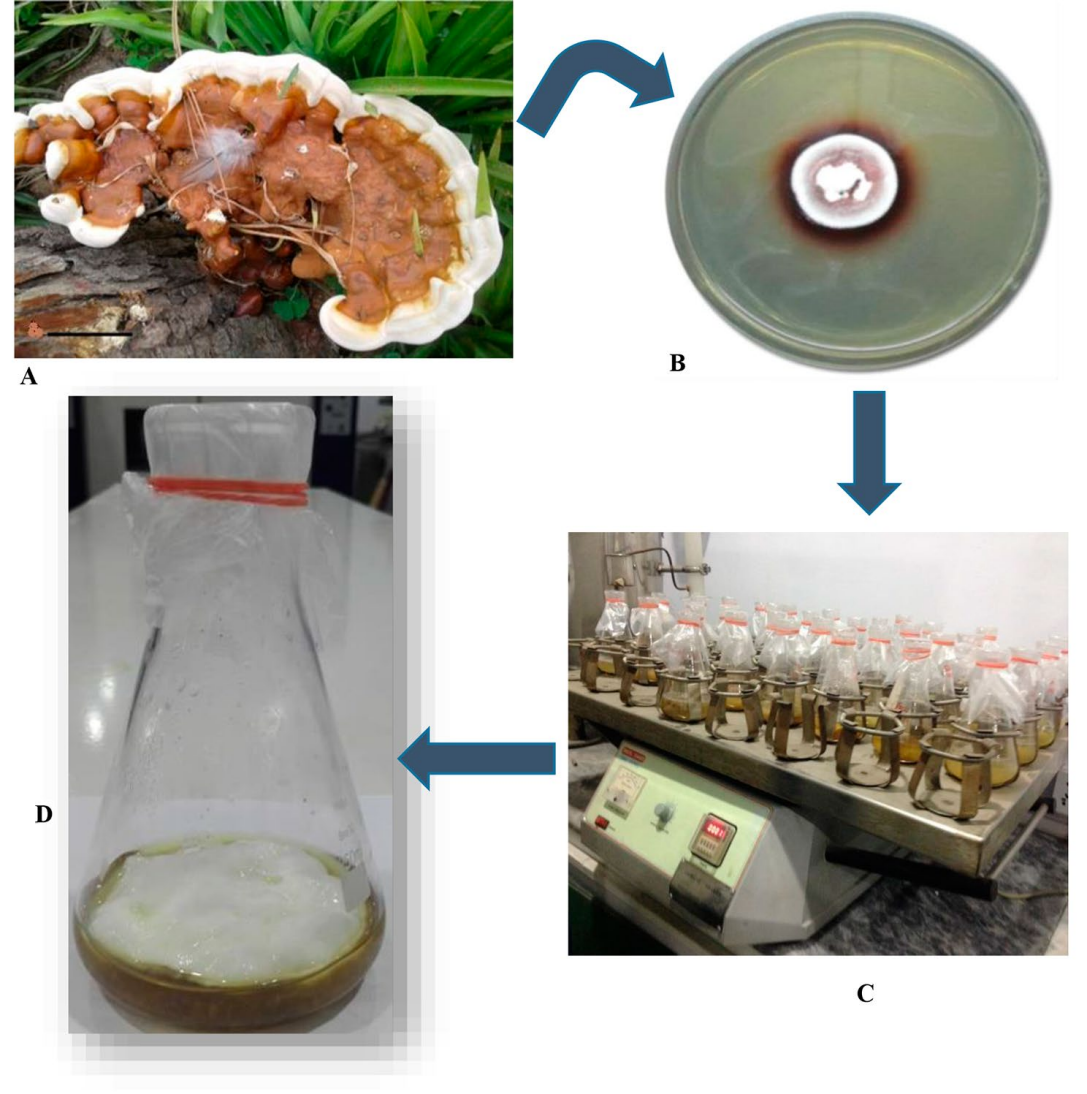
** A.** Basidiomata, **B.** Brown Oxidative Zone of Laccase, **C.** Quantitative estimation of **“Extracellular Laccase”** in shake flasks, **D.** Mycelia in flask for microscopy

The laccase producing *G. multistipitatum* (Fig. 2A) was preliminarily screened to produce the reddish brown zone on MEA plates (Fig. 2B C) contained guaiacol indicator. This exhibited a biggest reddish brown colored zone around the colony after 7 days of incubation at 27 °C.

### Quantitative laccase production

#### Statistical optimization of aeration/agitation on laccase production (by RSM)

RSM is a robust method in prediction of non-linear relationship between the variables and responses. Central composite design (CCD) used to evaluate the interactive effect of agitation rate, agitation time, and stationary time on production of laccase. Three factors at 5 levels of value (Table 1) resulted in 10 design experiments (Table 2) were presented in this study. For model validation, comparison of predicted and observed values of response variables (RS) also shown in this work (Table 3).

**Table 1:** Parameters range and independent variable levels

Sr No.	Range and levels	Variables		
		Agitation time (h)	Agitation rate (rpm)	Stationary time (h)
1	−√2	16	50	7
2	-1	21	100	10
3	0	27	150	13
4	1	33	200	16
5	+√2	37	235	18

**Table 2:** “Central composite rotary design matrix” with experimental values (agitation time, agitation rate, and stationary time)

Run	Agitation Time (h)	Agitation Speed (rpm)	Stationary Time (h)	Laccase Activity (U/L)
1	33	200	16	17.23 × 10^5^ ± 0.22
2	21	200	16	13.79 × 10^5^ ± 0.24
3	27	150	13	19.14 × 10^5^ ± 0.28
4	33	100	16	16.88 × 10^5^ ± 0.22
5	27	150	13	19.34 × 10^5^ ± 0.25
6	27	150	18	18.02 × 10^5^ ± 0.31
7	27	150	13	19.18 × 10^5^ ± 0.22
8	21	100	16	13.55 × 10^5^ ± 0.25
9	27	150	13	19.18 × 10^5^ ± 0.22
10	27	150	13	19.04 × 10^5^ ± 0.32

**Table 3:** Assessment of predicted and observed results of response variables obtained during model validation

Sr. No.	Agitation time (h)	Agitation rate (rpm)	Stationary time (h)	Predicted (× 10^5^ U/L	Obtained (× 10^5^ U/L	Variation (%)
1	33	50	10	12.78	12.82	1.3
2	27	100	16	15.38	15.89	3.77
3	27	150	13	19.16	19.44	2.21
4	31	200	13	16.78	17.56	3.06
5	31	235	13	8.28	9.45	1.1

#### Optimization of agitation speed for Laccase Production

**Fig. 3:**
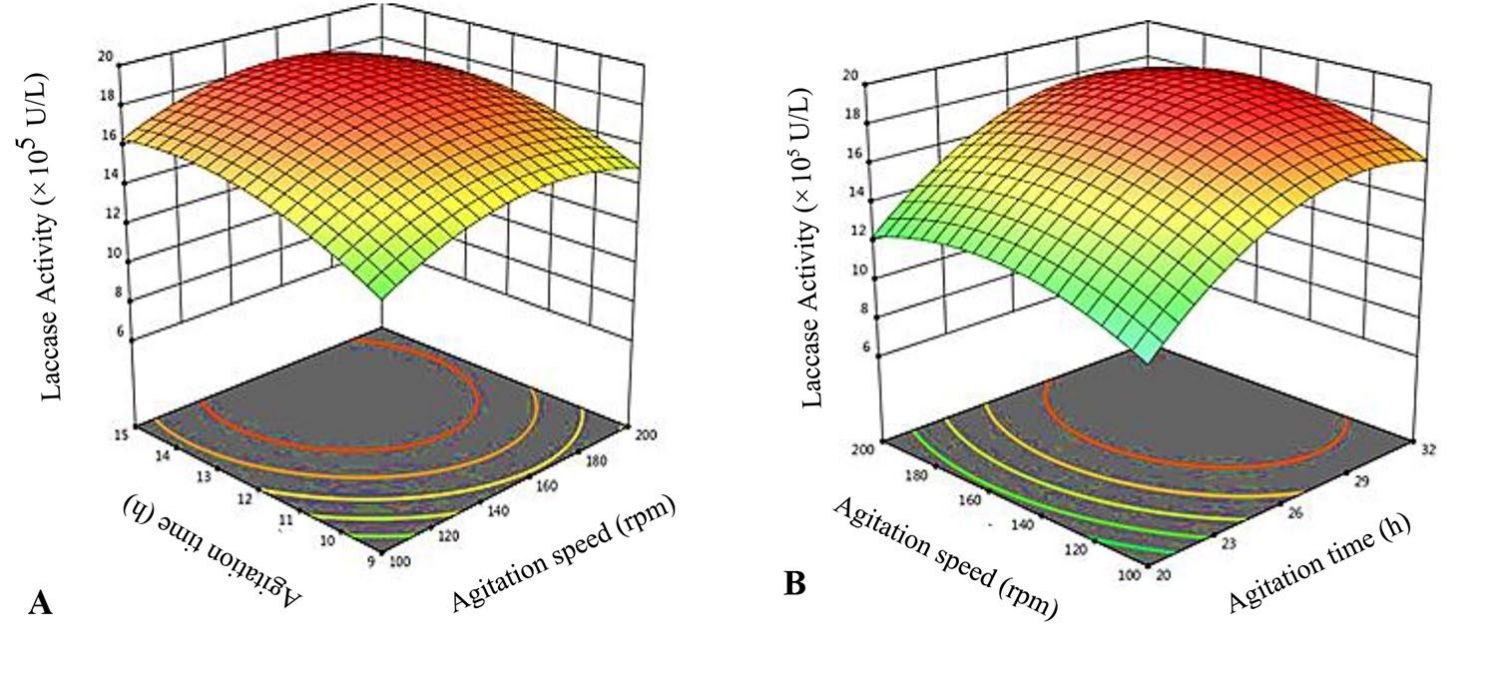
** A.** The 3D graphs showing interaction of agitation time and stationary time after agitation speed. **B.** Interaction of agitation time and agitation speed (laccase activity is represented as× 10^5^ U/L.

RSM sketched the non-linear relationship between variables and responses. CDR used to study the effects of agitation time, agitation speed (rate), and stationary time on production of laccase. Three factors value at five levels were presented in Table 1. The interaction between two variables, when 3rd at its optimum level was presented by contour plots, designed by using Design-Expert 10.0.5 (Fig. 3A, B). The plot response was shown at the mid point corresponding to laccase that affected by variation of two factors at a time “(kept third at level 0)”.

The growth and laccase secretion of fungal cultures depended on the agitation rate. In this study, the effect of speed at which rotating flasks influenced the growth of *Ganoderma* species to secrete laccase at 50, 100, 150, 200 and 235 rpm (Fig. 2C) was measured daily up to 15 days.

This was observed during the study that slow agitation of broth formed the filamentous mat of mycelium, due to constraint supply of O_2_ between fungal mycelium and medium. Mycelia’s shape and form was influenced by cultural conditions. In submerged phase, the mycelium of the *Ganoderma* species was scattered, formed mats, or sheared (Fig. 2D). At 200 rpm, the enzyme production was sharply decreased, whereas fungal growth increased considerably.

After fourth day of dynamic growth, the shake flask was settled at 50 rpm for 7–10 days, which increased the laccase activity in the culture broth at 25 °C. The rpm 50 was also suitable for secretion of laccase (12.82 × 10^5^ U/L) from mycelium. The species mycelium released maximum level (19.44 × 10^5^ ± 0.28 U/L) of laccase at 150 rpm (Fig. 3A). The higher laccase was released on seventh day at 100 to 150 rpm.

The 150 rpm was an excellent rotational speed for maximum secretion of laccase from mushroom mycelium in culture broth of shake flasks. The yield of laccase was increased, when flasks were switched from 100 rpm to 150 rpm. This concluded that greater than 150 rpm attributed to shear the mycelium biomass, which was detrimental for mushroom mycelium. Thus determined in this work that *Ganoderma* species are obligate aerobes demanded further parameters to secrete maximum laccase except rpm.

This study first time explored the inducing effects of aerobic conditions on *Ganoderma* sp. nov. for maximum laccase production. This is an alternative to time-consuming, costly and energy intensive techniques.

### Estimation of DO

The 150 rpm provided the continuous flush of oxygen from the environment. During laccase synthesis, DO was 65% in concentration at the end of “27 h” incubation. Later on, 13 h of static incubation lead to micro aerobic environment and DO concentration become reduced (44% DO) resulted an increase in laccase production. On another hand, comparison to 37 h of aerobic incubation, the DO was 58.35% at 150 rpm. The laccase production decreased, when complete anaerobic conditions occupied in flasks maintained for 13 h after 27 h of aerobic incubation at 150 rpm (Fig. 3B). The maximum yield of ganodermal laccase 19.44 (×10^5^ U/L) was achieved.

Agitation frequency provided the adequate heat transfer, mixing, mass and improved DO in culture medium. At lower agitation, the insufficient oxygen in flask medium usually affect the growth of microbes, although higher agitation sometimes lower the enzymes production as well. The maximum rotational speed develop a shear force among the cells in flask medium and the secretion dropped due to cell damages resulted from cell collision. Shear forces also given several effects on the ganodermal cells. This lead to morphological changes of the mycelium by damaging the internal and external cell structures, variation in growth, and yield formation. Consequently, the optimal rotational speed was necessary to determine the maximal enzyme production in submerged fermentation by using a flask system.

**Fig. 4:**
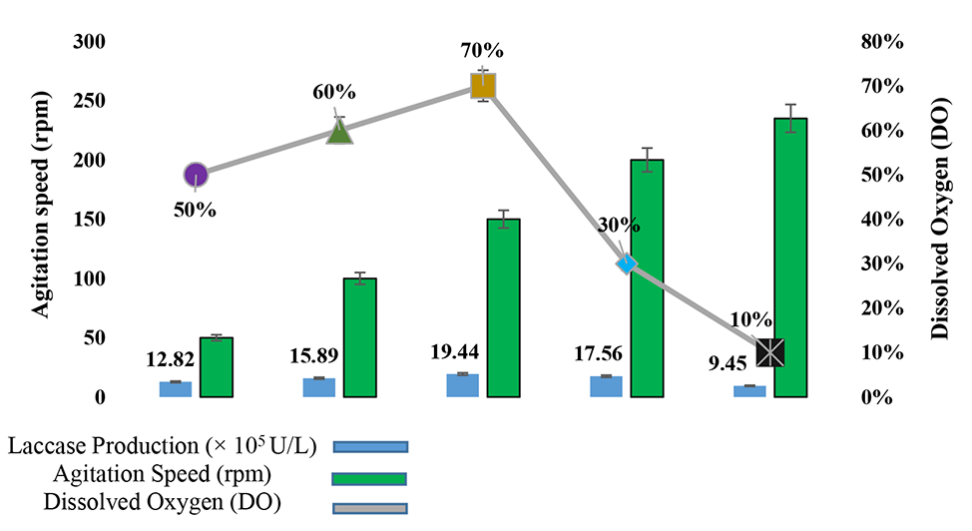
The percentage of DO interacted with agitation speed and laccase production (× 10^5^ U/L)

The 150 rpm provided a continuous flush of oxygen from the environment, when the laccase production started at 150 rpm, the DO concentration was 70% at the end of 27 h incubation. The DO was 30% at 50 rpm, also facilitated the laccase production. The laccase production decreased, when the complete anaerobic conditions prevailed e.g. static condition. The higher yield of laccase (19.44 × 10^5^ U/L) was achieved at 150 rpm. The production of laccase was slow down at 200 and 235 rpm, when mycelium shearing started (Fig. 4).

Limited O_2_ was supplied to the mycelium of culture medium at lower agitation speed, whereas higher speeds sometimes lower the enzymes production. Higher agitation speed developed the shear forces among the suspended fungal cells of culture medium and the production rate dropped down due to cell collision resulted cell damages. Shear forces also given multiple effects on the fungal cells. This force also caused the changes on fungal growth, morphology, damaging the external and internal cell structures, and yield formation.

### Observations under compound and scanning electron microscope

**Fig. 5:**
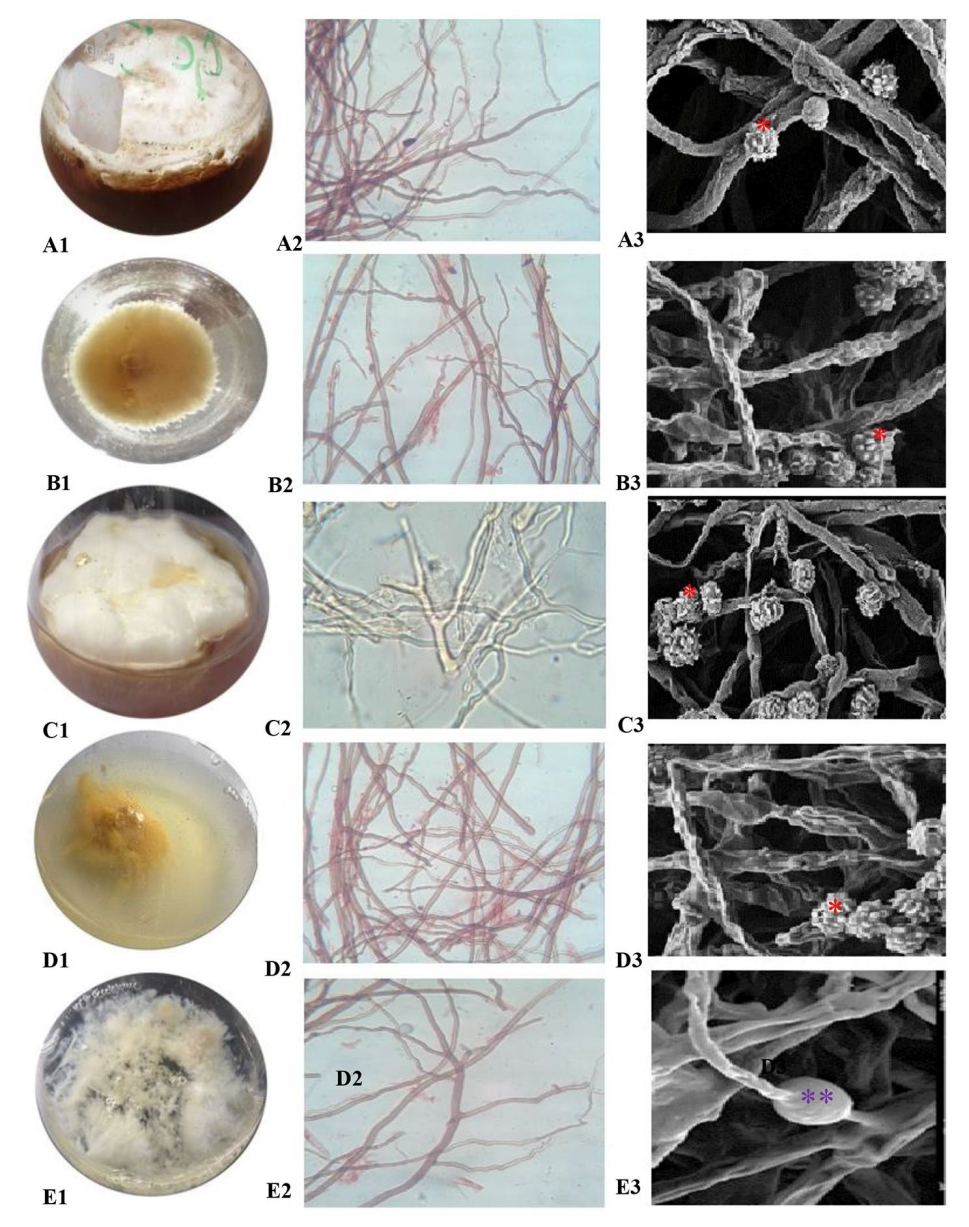
Mycelium morphology in rotating shake flasks of *Ganoderma multistipitatum* sp. nov. (A1-50 rpm, B1-100 rpm, C1-150 rpm, D1-200 rpm, E1-230 rpm), CM images (A2 to E2), and SEM images (A3 to E3) [asterisk indicate chlamydospores (*) and spores (**)]

Compound microscopy (Fig. 5A2 to E2) and SEM (Fig. 5A3 to E3) was carried out for clearer view of mycelia. The micrographs shown the branched and cylindrical shape mycelia with spores and chlamydospores This condition occurred to the fungal culture agitated at low agitation speeds, i.e., at 50 (Fig. 5A1), 100 rpm (Fig. 5B1), 150 rpm (Fig. 5C1), 200 rpm (Fig. 5D1), and 230 rpm (Fig. 5E1). Although at higher agitation speeds (200 rpm), the fungal culture exhibited less branching system. There was no formation of fruiting bodies at 200 rpm.

The mycelium grown in lower conditions (50 and 100 rpm) showed branched mycelia with numerous basidiomata (fruiting bodies) (Fig. 5B2 to 5B3). At 150 rpm, formation of hyphae with maximum number of fruiting bodies were observed with tight packing structures (Fig. 5C2 to 5C3). However, agitation speed of 200 and 230 rpm exhibited the shear effects on branched mycelia with minimum number of fruiting bodies (Fig. 5D2 to 5E3). The fungal growth was best at the agitation of 150 to 200 rpm, which exhibited smoother, not well rounded hyphae and highly dense branched mycelia. The formations of spores were also seen at 230 rpm.

In this work, experimental design and response surface methodology was used to optimize the laccase production from *G. multistipitatum* in submerged fermentation. Response surface analysis showed that 150 rpm was an optimal condition for maximum laccase (19.44 × 10^5^ ± 0.28 U/L) secretion by *G. multistipitatum*. Under these conditions, the predicted value of laccase activity was in good agreement with the laccase activity obtained experimentally. The experimental design and response surface methodology was successfully used to determine the effects of parameter on substantial production of maximum laccase. Agitation speed of the medium and its interaction with dissolved oxygen affected the laccase production and mycelium morphology. In this study, the obtained result of surface methodology for optimal condition for laccase production was successfully validated by performing an experiment under similar conditions.

## Discussion

Laccase is an expensive health caring enzyme extensively used in food, bread dough, fruit juices for flavor, freshness, volume, texture, and nutritional values of health oriented products [27]. Consumers perceive laccase as a natural and non-toxic food component over the chemicals used for food-processing. Interestingly, laccase found in epithelial cells of human intestines. So, genetic variation in the gene coding for human laccase has been associated with the risk of Crohn’s disease, leprosy, ulcerative colitis, and juvenile idiopathic arthritis [28]. It is worth noting that the need of laccase for rapid population is increasing day by day. So to fulfil the demands of living beings, researchers are exploring the stronger candidates for laccase with maximum redox potential. Mostly, laccase of industrial and healthcare interests are produced by wood rotting fungi. The fungal laccase possessed the highest redox potential than plant, bacteria, insects, and animals. The natural origin, non-toxicity, and mild operating conditions on fungi to release their enzymes have stimulated a plethora of research to opt the optimize conditions (by submerged fermentation) for maximum production of laccase to fulfil the increasing demands.

Growth of hyphae in form of branches called mycelium. In submerged flask, the fungal culture knitted like mycelium (thick mat, minimum thick mat, free mycelia, and freely spread mycelia throughout the culture medium [29]. The morphology of mycelium in submerged fermentation phase implemented the good effects on the productivity and metabolites production as well. Agitation/rotation has effect on enzymes production of the microorganisms.

Increased aeration and dissolved oxygen of the culture medium was sufficient to enhance the laccase production. At 200 rpm, the enzyme production dropped even though the fungal growth increased considerably. The suitable aeration system and nutrients caused the fungus to proliferate well, whereas shear forces and cell damage had negative effect on enzyme production [29]. However, the various fungal species demonstrated the different optimal agitation speeds require to maximize the enzyme yield in submerged fermentation.

Agitation speed and dissolved oxygen concentration on pectinase production are important leading factors to successful progress of fermentation. Sufficient dissolved oxygen in medium is important for transfer characteristics of microbial cells [30]. Agitation provides adequate rotational speed, facilitate in mixing of nutrients, heat, and mass transfer. Rotational speed creates the shear forces, which leads to morphological changes and eventually damage the cells and associated structures [29].

Higher rpm may indicate the improved oxygen transfer to *G. lucidum* mycelium in fermenting broth according to Yang and Liau [31]. Additionally, they concluded that speed greater than 150 rpm caused the mycelium biomass to be sheared, which was bad for mushroom mycelium. Tiny pellets were formed in shake flasks due to continuous stirring of fermentation medium for maximum enzyme secretion [32]. For the greatest laccase synthesis, Rodrigues et al. [33] cultured *G. lucidum* fungal discs on PDA medium for 8 days at 150 rpm. During an agitated situation of submerged culture, the parent fungal colonial mat of *Polyporus grammocephalus* was divided into numerous smaller colonies known as “daughter colonies” [34].

Industrial applications get benefits from the highest laccase production in shortest time, whereas fungal species require a longer laccase production cycle [35]. Researchers also need a longer fermentation duration of 14 or 20 days to get the highest laccase level [36]. On third day of incubation, the fungal mycelium biomass filled the liquid medium by a self-immobilizing biomembrane for maximum laccase secretion. This hypothesis on *Gaonderma lucidum* was refuted by our findings [37]. On day 7 of our investigation, the mycelium biomass of *Ganoderma* completely covered the broth. The reported laccase activity by submerged fermentation was 27 UL^− 1^ from *Ganoderma* sp., 80 UL^− 1^ from *G. australe*, and 120 UL^− 1^ from *Ganoderma* sp. En3 [38]. According to one school of thought, the development rate of fungus mycelium slow down on day 6 and referred as “deceleration phase”, because thicken bio membrane prevented the higher cells of mycelium from absorbing the most nutrients from the broth. The mycelium exhausted due to lack of nutrients, this phase allow the entrance of bacteria and other microbes, which forced the mycelium to turn into “death phase” on days 10–15. At this stage, nutrients are entirely scarce, and mycelium biomasses thereafter become feeble and exhausted [37].

This study was in line with previous researches, which indicated that laccase was very low during the secondary growth phase on day 5, but reached its maximum value (20 U/ml) on day 9 to 13, which was lower than the value found in typical reported strains of *Ganoderma* (4-100 U/ml) [39]. Laccase formation from *G. lucidum* was influenced by media composition and time [33]. On the other hand, *G. lucidum* showed the highest activity on sixth (707 U/L) and seventh (785 U/L) day, while lowest on eighth day (607 U/L). On 6 to 8th days, the laccase activity was 707 U/L, 785 U/L, and 607 U/L, respectively [38].

Overall, aeration and agitation was a simpler technique in fermentation system, where minimum operating cost required. Thus, agitation speed control living system of fungal mycelium for maximum laccase production.

## Data Availability

The data set generated and analyzed during the current study is available from the corresponding author on personal request. The consensus were deposited to GenBank under accession number ON032992, ON032991.

## List of abbreviations

ITS: Internal Transcribed Spacer
CTAB: Cetyl Trimethylammonium Bromide
BLAST: Basic Local Alignment Search Tool
NCBI: National Center for Biotechnology Information
MAFFT: Multiple Alignment using Fast Fourier Transform
MEGA: Molecular Evolutionary Genetics Analysis
DO: Dissolved Oxygen
RSM: Response surface methodology
ML: Maximum likelihood
